# Correction to “Population Genomics Informs Conservation Strategies for Critically Endangered *Kokia* Species in Hawai‘i”

**DOI:** 10.1002/ece3.73836

**Published:** 2026-06-10

Ning, W., E. Kayal, J. F. Wendel, et al. 2026. “Population Genomics Informs Conservation Strategies for Critically Endangered Kokia Species in Hawaiʻi.” *Ecology and Evolution* 16, no. 4: e73104. https://doi.org/10.1002/ece3.73104.

The below affiliations should have been added to the authors Ehsan Kayal and Zenaida V. Magbanua, respectively.

Department of Biology, University of Wisconsin–River Falls, River Falls, WI 54022, USA.

Department of Biochemistry, Nutrition, and Health Promotion, Mississippi State University, Mississippi State, MS 39762

We apologize for this error.

