# Supplementary material for: Population Genomics Informs Conservation Strategies for Critically Endangered Kokia Species in Hawaiʻi
Source: Ecol Evol. 2026 Mar 31;16(4):e73104. doi: 10.1002/ece3.73104 (PMC13106990; doi:10.1002/ece3.73104)

Supplementary Figure 1

Interspecific and intraspecifc LEA analysis for three extant Kokia species, with sample information labeled.

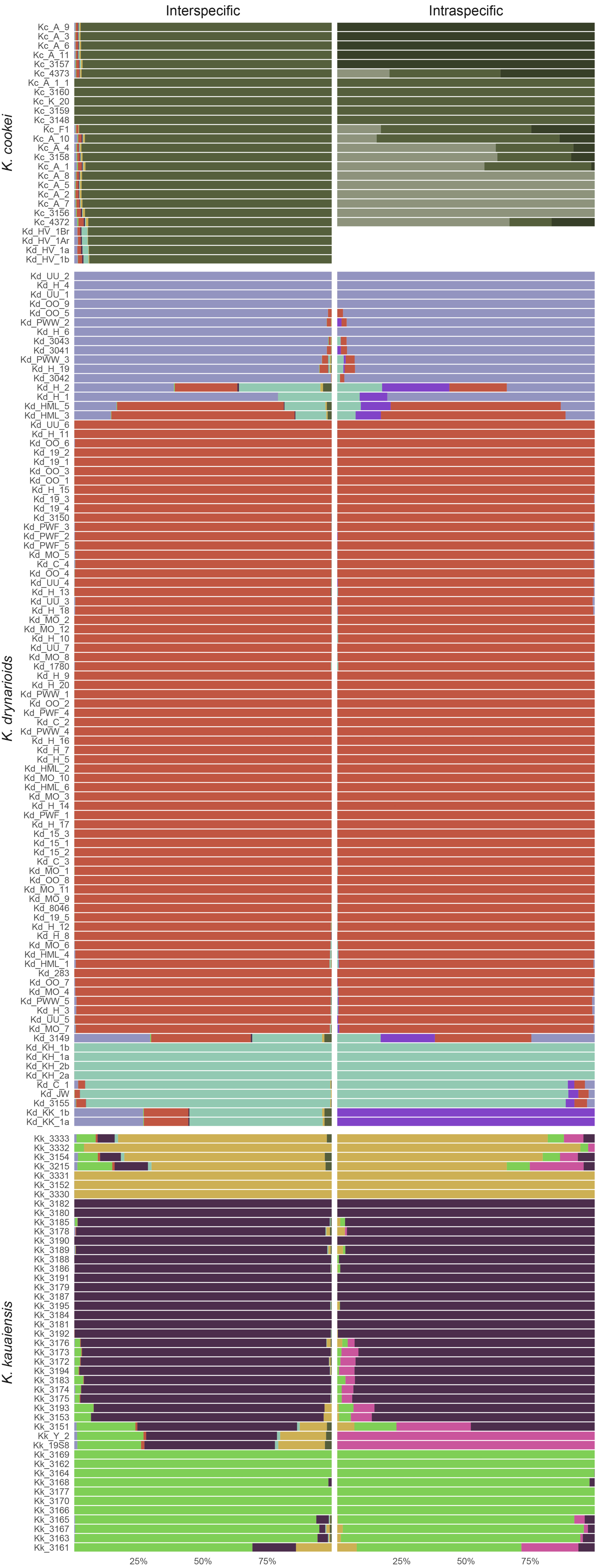

Supplement: Supplementary file 1 — Figure S1: Interspecific and intraspecific LEA analysis for three extant Kokia species, with sample information labeled. [file ECE3-16-e73104-s002.pdf]
