# Supplementary material for: Population Genomics Informs Conservation Strategies for Critically Endangered Kokia Species in Hawaiʻi
Source: Ecol Evol. 2026 Mar 31;16(4):e73104. doi: 10.1002/ece3.73104 (PMC13106990; doi:10.1002/ece3.73104)

Supplementary Figure 2

Species-specific PCA (A) and LEA (B) analysis for K. cookei including the newly identified HAVO tree (Kd_HV_1Ar). The HAVO sample is identified in the PCA using blue (versus red) coloring, reflecting its original species assignment.

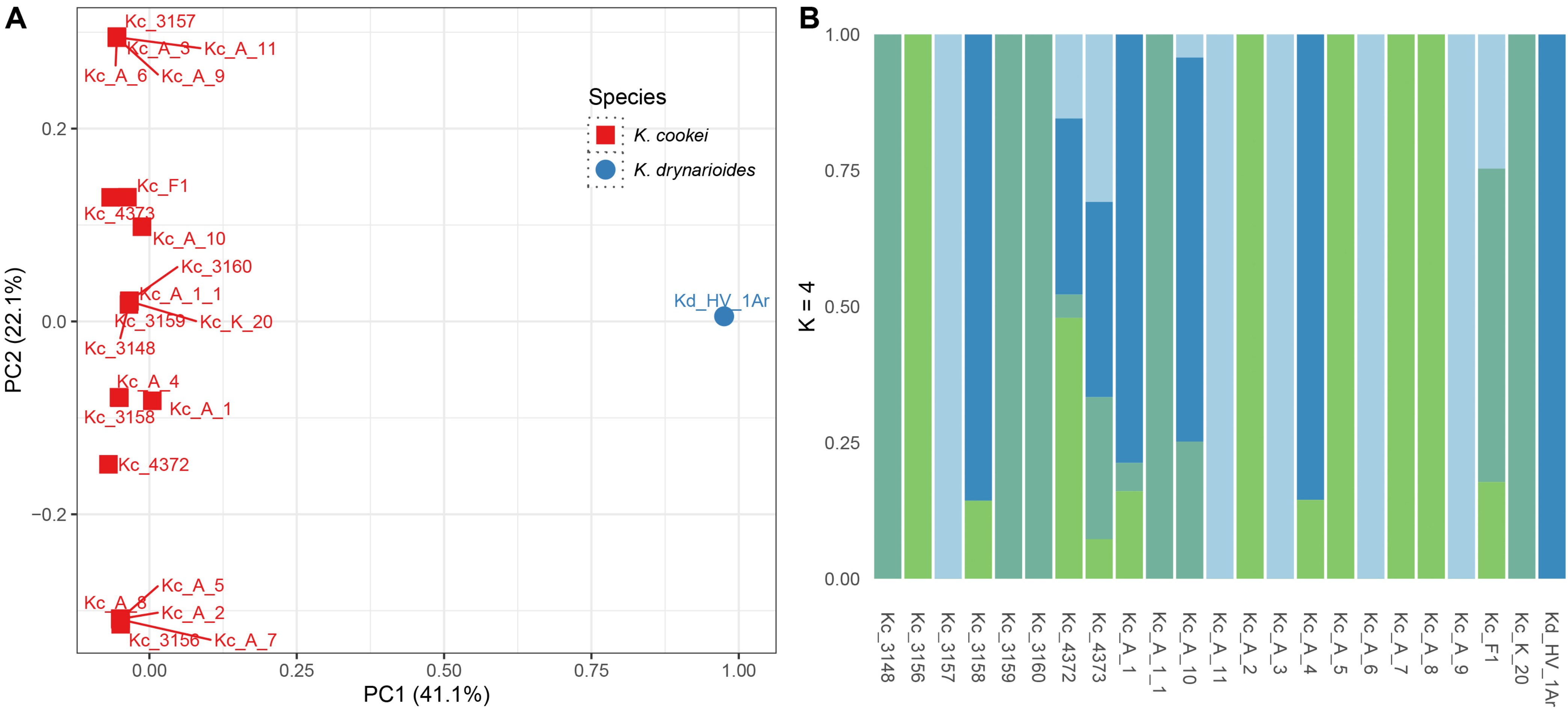

Supplement: Supplementary file 2 — Figure S2: Species‐specific PCA (A) and LEA (B) analysis for K. cookei including the newly identified HAVO tree (Kd_HV_1Ar). The HAVO sample is identified in the PCA using blue (versus red) coloring, reflecting its original species assignment. [file ECE3-16-e73104-s005.pdf]
