# Supplementary material for: Population Genomics Informs Conservation Strategies for Critically Endangered Kokia Species in Hawaiʻi
Source: Ecol Evol. 2026 Mar 31;16(4):e73104. doi: 10.1002/ece3.73104 (PMC13106990; doi:10.1002/ece3.73104)

Supplementary Figure 3

LEA populations by site for K. drynarioides, both for the interspecific and intraspecific analyses.

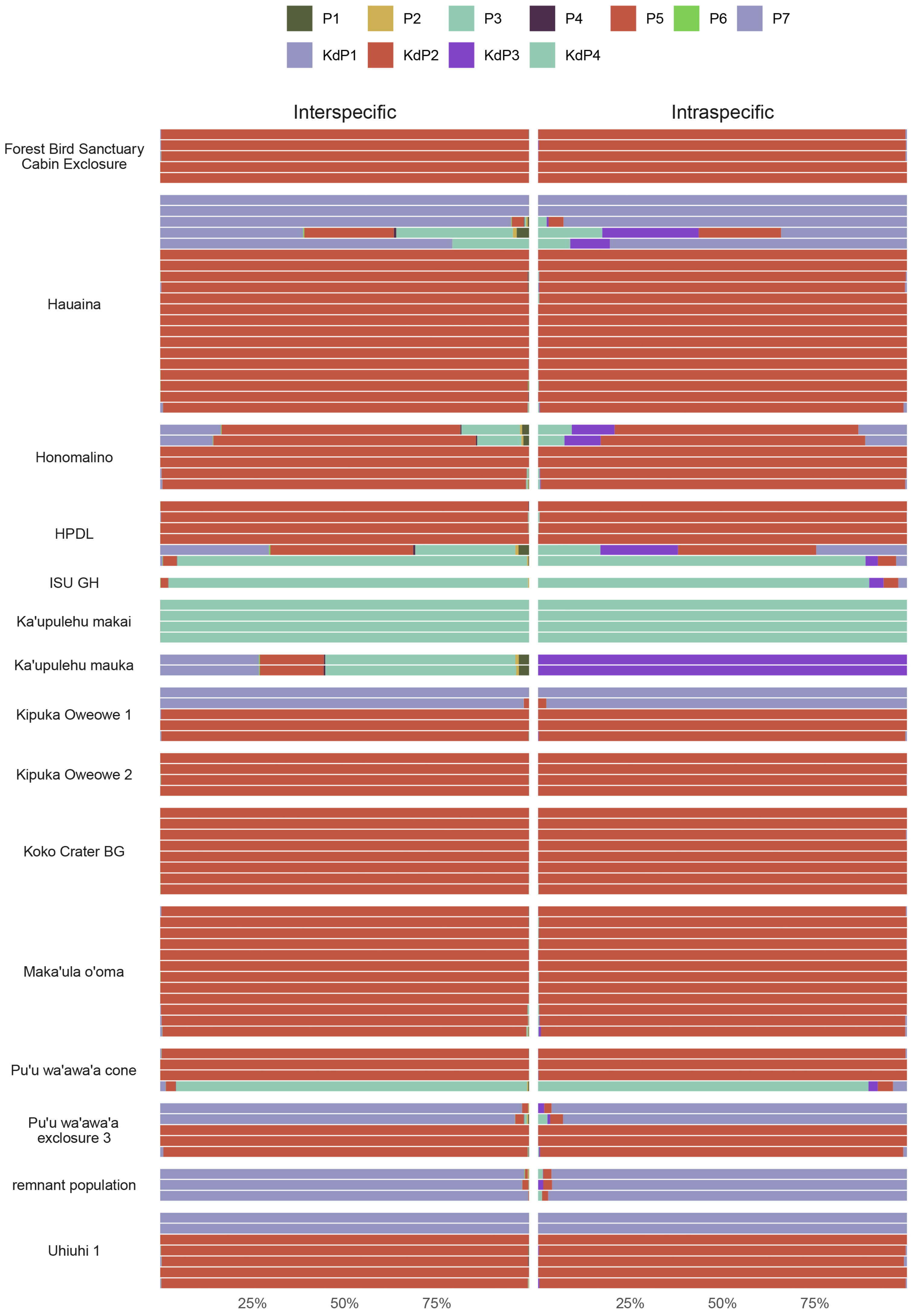

Supplement: Supplementary file 3 — Figure S3: LEA populations by site for K. drynarioides , both for the interspecific and intraspecific analyses. [file ECE3-16-e73104-s001.pdf]
